# Heterogeneities in Cognitive and Socio-Emotional Development in Children With Autism Spectrum Disorder and Severe Intellectual Disability as a Comorbidity

**DOI:** 10.3389/fpsyt.2019.00508

**Published:** 2019-07-19

**Authors:** Marie-Anna Bernard Paulais, Camilla Mazetto, Eric Thiébaut, Maria Clara Nassif, Maria Thereza Costa Coelho De Souza, Ana Paula Stefani, Romuald Blanc, Maria Pilar Gattegno, Fethia Aïad, Nadia Sam, Lina Belal, Laid Fekih, Kelley Kaye, Yves Contejean, Jaqueline Wendland, Catherine Barthélémy, Frédérique Bonnet-Brilhault, Jean-Louis Adrien

**Affiliations:** ^1^Laboratory of Psychopathology and Health Processes (EA n°4057), Institute of Psychology, University of Paris, Paris, France; ^2^Psychology Office ESPAS-IDDEES, Pont-Ste-Maxence and Bordeaux, France; ^3^CARI Psichology and Education, São Paulo, Brazil; ^4^Institute of Psychology, University of São Paulo, São Paulo, Brazil; ^5^Lorrain Laboratory of Psychology and Neurosciences of Behaviors’ Dynamics (2LPN), University of Lorraine, Nancy, France; ^6^UMR 1253, iBrain, University of Tours, INSERM, Tours, France; ^7^Psychology Office ESPAS-IDDEES, Bordeaux, France; ^8^Language, Cognition and Interaction Laboratory, University of Blida 2 Lounici Ali, Blida, Algeria; ^9^Faculty of Social and Human Sciences, University Abdelhamid Ibn Badis, Mostaganem, Algeria; ^10^Laboratory of Psychometric and Its Applications, University Abou Beker Belkaid, Tlemcen, Algeria; ^11^Child Psychiatry Department of Sainte Anne Hospital, CREDAT, Paris, France

**Keywords:** autism spectrum disorder, intellectual disability, Comorbidity, heterogeneities, cognitive and socioemotional developmental profiles, The Social Cognitive Evaluation Battery

## Abstract

**Introduction:** Intellectual disability (ID) is frequently associated as a comorbidity in autism spectrum disorders (ASD). This study investigated a) how similar the heterogeneity in the cognitive and socio-emotional developmental profiles was for children with ASD and ID, b) the difference between the subjects’ profiles and those of typically developing children (TD) matched for developmental levels, c) the skills existing with the lowest and highest developmental levels, and d) the relationship between developmental profiles in ASD and the severity of autism, ID, and the overall developmental level.

**Participants:** The sample was comprised of 119 children (101 boys and 18 girls) who ranged in chronological age (CA) from 21 months to 14 years (*M* = 5 years 2 months; SD = 2 years 6 months) with developmental levels lower than 24 months. They came from three countries (France = 40, Brazil = 40, and Algeria = 39). The control group was comprised of 40 TD children from these same countries who ranged in CA from 4 to 24 months (*M* = 1 year 3 months; SD = 5 months). The ASD diagnosis was carried out according to International Statistical Classification of Diseases and Related Health Problems-10th Edition (ICD-10), Diagnostic and Statistical Manual of Mental Disorders, 4th Edition, Text Revision (DSM-IV-TR), Diagnostic and Statistical Manual of Mental Disorders-5th ed (DSM-5) criteria and the Childhood Autism Rating Scale (CARS).

**Measures:** Children were tested using the Social Cognitive Evaluation Battery (SCEB; Adrien, 2007) by trained psychologists from public and private institutions specialized in the diagnosis of autism and interventions in this field. The SCEB explores 16 functional abilities, in both cognitive and socio-emotional areas, and allows the calculation of domain and area developmental levels and heterogeneity indices for the global, cognitive, and socio-emotional areas.

**Results:** Children with ASD developmental profiles show very high heterogeneity as opposed to TD children. Regardless of the country of origin, there are similarities between the heterogeneous cognitive and socio-emotional developmental profiles of the children with ASD, whose profiles are characterized by lower developmental levels of language and vocal imitation skills, and a relationship between these developmental heterogeneities and the degree of severity of autistic symptomatology, intellectual disability, and overall development level. The implications of this study are presented for clinical assessment and intervention purposes in ASD and ID.

## Introduction

Autistic spectrum disorder (ASD) is characterized by disturbances in social interaction, communication and repetitive activity Diagnostic and Statistical Manual of Mental Disorders-5th ed. (DSM-5) (1). The continuum of autism is identified and diagnosed by assessing not only the nature of the disorder (including hyper- and hypo-sensory reactivity) but also its severity. Intellectual disability (IQ less than 70), previously referred to as “mental retardation” (2) and with a wide variability in profiles (3) affects more than half of the children with autism (4, 5) but Christensen et al. (6) showed that the percentage of children with an intellectual disability (ID) varied widely across 9 out of 11 geographic areas, ranging from 20 to 50%, and significantly more girls had ASD associated with ID (37%) than boys (30%). Using the criteria of DSM-5, Brown et al. (7) reported IQ prevalence and indicated that about 40% of the ASD population are likely to present ID. In the present study, differences and similarities in the cognitive and socio-emotional developmental profiles of children with ASD and comorbid ID as compared with those of typical children were examined across three countries (France, Brazil, and Algeria).

Clinical heterogeneity is a notable characteristic of ASD. Lombardo et al. (8) noted that this heterogeneity is present at several levels of analysis such as genetics, neural systems, cognition, behavior, and development, as well as in clinical features (e.g., response to treatment, outcome). From a developmental psychopathology approach, the heterogeneity in question encompasses different variations between and within individuals relative to their developmental, cognitive, and behavioral characteristics. Between individuals with ASD, developmental heterogeneity may be characterized by some different developmental trajectories (9) and outcomes relative to verbal and nonverbal abilities (10) and intellectual and socio-adaptive development (11). Within individuals, heterogeneity may be defined at a first level in terms of functional dysregulation in using mental representation, symbolic play, social communication, and sensory-motor abilities (12–21). Moreover, at a second level, heterogeneity is defined by some atypical differences in developmental stages between several abilities, explained by the changes in the timing and rates of infant and child development, corresponding to the unevenness or developmental heterochrony (22).

This type of heterogeneity was already attested in children with autism by a large number of studies centered on verbal and nonverbal communication, sensorimotor, and cognitive functions profiles, showing evidence of atypical patterns, discrepancies, and unusual correlates between developmental levels of various cognitive and socio-communicative skills (23–28). Moreover, in the intellectual domain, the profiles of children with autism are usually characterized by a discrepancy between nonverbal abilities and verbal abilities (NVIQ > VIQ). This discrepancy lessened with age in children with functional language and overall cognitive abilities in the mildly impaired range or above (29). Moreover, it was specifically associated with a high level of symptoms in the social domain (30) and correlated to the intensity of autistic symptomatology (31). Most of these works described this developmental heterogeneity in children with ASD whose developmental ages were above 2 years of age (29, 32, 33). However, only a few studies describe the cognitive and socio-emotional profiles of children with developmental levels under 2 years of age with a moderate or severe ID. Thiébaut et al. (34) showed evidence of heterogeneous developmental cognitive and socio-emotional profiles in these children with strong inter- and intraindividual variability and developmental delay contrary to what is observed in children with ID without ASD (35).

Cross-cultural studies in ASD are still recent and have focused on different ways of thinking and understanding autism as a disorder (36, 37) and on the diversity of symptoms, characteristics, or traits of ASD using screening tools (38–40) and on social skills interventions (41). The heterogeneity of the developmental profile of children with ASD across several countries in North and South America, North Africa, and Europe (42–44) was pointed out. While no significant differences in overall, cognitive and socio-emotional development levels between two groups of young (1 year and 6 months to 3 years of age) and older children (8–14 years of age) with ASD from the same countries were observed, the youngest group of children exhibited a greater socio-emotional heterogeneity (45). However, the samples of children in each of the seven countries were small and very different from one another. Therefore, in this study, using larger and similar-sized samples of children, we sought to examine whether the heterogeneity of the cognitive and socio-emotional developmental profile in children with ASD and comorbid ID from three countries was different from typical children, had the same intensity, was independent of the country of origin, and correlated to the severity of autistic symptomatology and of ID and the overall developmental level.

## Method

### Participants

The total sample of participants with ASD included 119 children (101 boys and 18 girls; gender ratio = 5.61:1; mean age = 5 years 3 months, from 1 year 9 months to 14 years of age; SD = 2 years 6 months) from three countries, Brazil, Algeria, and France. Clinical data were collected for 40 Brazilian children with ASD at the CARI Psychology and Education Clinic and at the Centro Pró-Autista, both in the city of São Paulo, where children received a diagnostic assessment and a neuropsychological and developmental program (46). There were also 39 Algerian children who were referred by several private and public institutions providing behavioral, integrative, and psycho-educational interventions to children with autism. Clinical data for an equivalent sample of 40 French children with ASD were taken from databases of different clinical services and used in some previous studies (34, 42, 47). The French children with ASD were treated in the Child and Adolescent Psychiatry Department of the University Hospital Center Bretonneau in Tours, in the Psychology Offices ESPAS-IDDEES, and in the Child Psychiatry Department of Sainte Anne Hospital in Paris. As a control group, data from 40 children with typical development (TD) was gathered (Algeria *N* = 13, Brazil *N* = 13, and France *N* = 14) randomly from the same database. The subjects were recruited from public nurseries and in the professional or social environment of psychologists. All the children were assessed in the country where they lived and in their native language. In an analysis of variance, with four groups of 40 participants, the probability of detecting an average effect (48) at the α threshold of 0.05 is 0.98. For a correlational study, with 40 participants per group, the probability of detecting an average effect (*r* = 0.3) at the α threshold of 0.05 is 0.90. Groups of 40 participants would then seem sufficient to detect an average effect with reasonable success.

To obtain background information about each participant, information on age, gender, and diagnostic status (where, when, and by whom a diagnosis had been made) was used. The diagnosis for children younger than 3 years (*N* = 20) was confirmed some months or years later. All children included in our clinical sample were diagnosed with autism disorder according to the criteria of International Statistical Classification of Diseases and Related Health Problems-10th Edition (ICD-10) (49) and Diagnostic and Statistical Manual of Mental Disorders, 4th Edition, Text Revision (DSM-IV) and confirmed as ASD subjects based on DSM-5 criteria (1) and assessed with the Childhood Autism Rating Scale (CARS) (51). Intellectual disability (ID) diagnosis was established from developmental assessments with the Socio-emotional and Cognitive Evaluation Battery (SCEB) (47) for all the children, thus avoiding possible variations in the data due to the use of several different tests. Developmental Quotient (DQ) was calculated based upon the existence of very high correlations between the overall scores (not corrected for unreliability) of the SCEB and the psychomotor development ages calculated with the Psychomotor Development Scale of Brunet–Lézine Revised (52), a French adaptation of Gesell developmental scales (53), which included assessments on postural, verbal, nonverbal, and sociability domains.

The diagnoses of ASD and ID as a comorbidity were performed by child psychiatrists and psychologists experienced in ASD and other neurodevelopmental disorders. Demographic information of participants is shown in **Table 1**.

**Table 1:** Chronological age (year-yr and month-mth) in autism spectrum disorder (ASD) groups (*N* = 119) and typical development group (*N* = 40).

	Chronological age (years and months)			
Country	Mean	SD	Min.	Max.
Algeria (*N* = 39)	6 yr, 3 mth	2 yr, 3 mth	2 yr, 2 mth	12 yr, 4 mth
Brazil (*N* = 40)	4 yr, 4 mth.	1 yr, 2 mth	2 yr, 9 th.	6 yr, 7 mth
France (*N* = 40)	5 yr, 3 mth	3 yr, 3 mth	1 yr, 9 mth	14 yr, 0 mth
Total (*N* = 119: 101 male, 18 female)	5 yr, 3 mth	2 yr, 6 mth	1 yr, 9 mth	14 yr, 0 mth
**Typical development (** ***N*** ** = 40)**	1 yr, 3 mth	5 mth	4 mth	2 yr., 0 mth

Some differences (two-sided *p* value) appeared between ASD groups for chronological age [*F*(2, 115) = 6.44, *p* = 0.002]. The Brazilian group was slightly younger (from 4 to approximately 6 years of age) than the other two groups (from 5 to 14 years of age).

Although the children were recruited from a variety of settings, the developmental evaluation of each child was organized at the start for a diagnostic decision or for monitoring his/her evolution during overall and intensive psycho-educational care. Psychological evaluations were recorded in a written report. The study was carried out in accordance with official laws^1^ and standards of ethics, biomedical, and clinical research in France. In Algeria, it was done with the University Charter of Ethics and Deontology of the Algerian Ministry for Higher Education and Research^2^, and in Brazil, the study was approved by the National Commission for Research Ethics (CONEP) under the aegis of the Brazilian Ministry of Health^3^. All data were anonymized. Written and informed consent was obtained from the legal guardians who were assured of the noninvasive nature of the research and the confidentiality of the data. Furthermore, the systematic use of video recordings during evaluations was subject to written consent from the families.

### Material

The Childhood Autism Rating Scale (CARS) (51) was rated by trained psychologists experienced in ASD who carried out the developmental assessments of the children participating in the study. Rating was carried out at the end of the examination.

**Figure 1:**
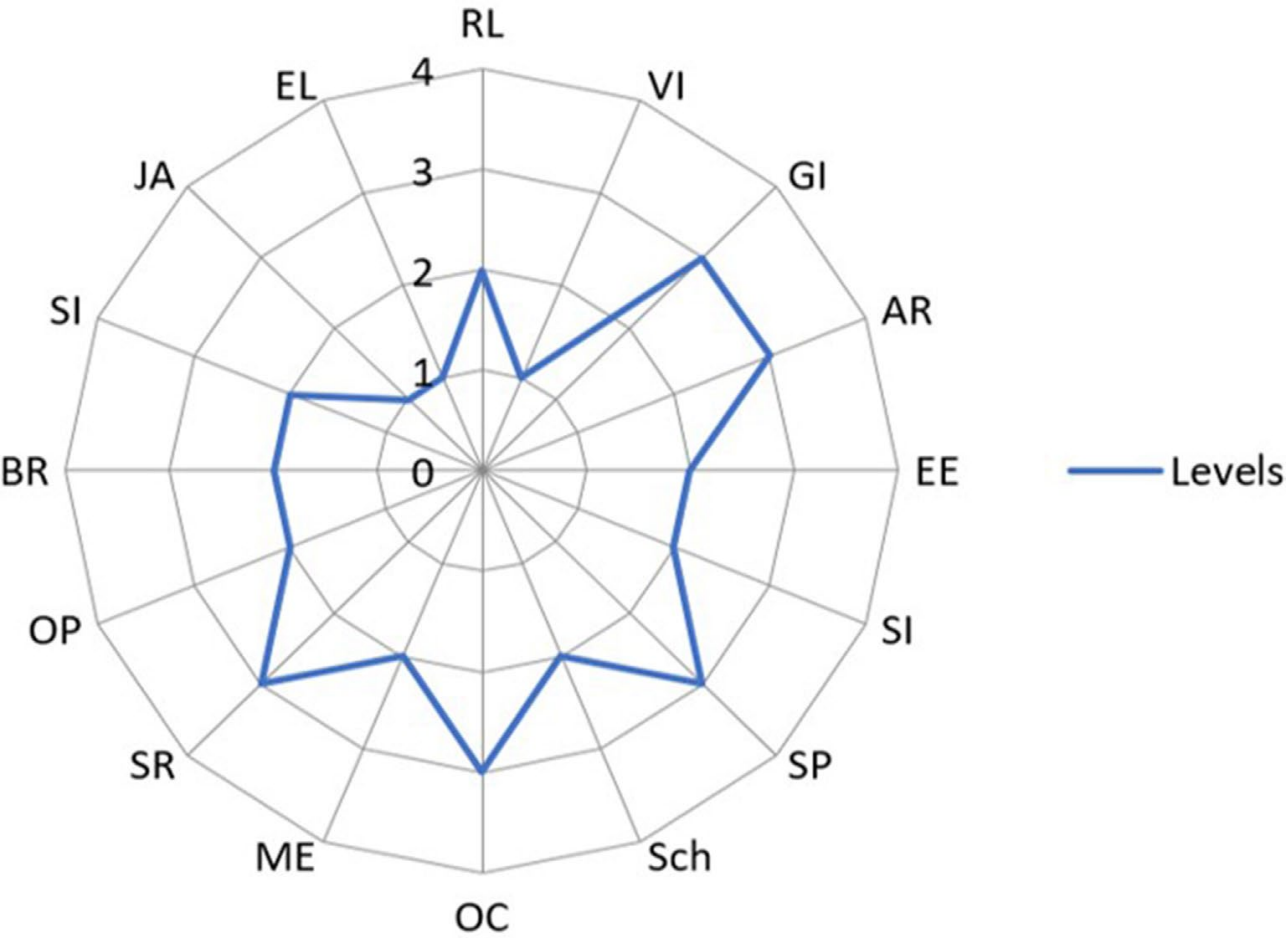
Example of a cognitive and socio-emotional developmental profile of a child with autism spectrum disorder (ASD) obtained from the Social Cognitive Evaluation Battery (SCEB). *Legend*: The four developmental levels (from 1 to 4) are represented by four concentric circles. Each domain of development is represented by a radius of the circles. *Socio-emotional domains*: Behavior Regulation (BR, level 2), Social Interaction (SI, level 2), Joint Attention (JA, level 1), Expressive Language (EL, level 1), Receptive Language (RL, level 2), Vocal Imitation (VI, level 1), Gestural Imitation (GI, level 2), Affective Relation (AR, level 2), and Emotional Expression (EE, level 2). *Cognitive domains*: Self-Image (SI, level 2), Symbolic Play (SP, level 3), Object relation schemata (Sch, level 2), Operational Causality (OC, level 3), Means–Ends (ME, level 2), Spatial Relations (SR, level 3), and Object Permanence (OP, level 2).

All assessments of the development of children in the study were performed using the Socio-emotional Cognitive Evaluation Battery (SCEB) (47), an instrument specifically created for the examination of preschool and school-aged children with autism and intellectual disability and recommended by the French High Health Authority (54, 55). This battery can be used to examine children who have a developmental level between 4 and 24 months and to assess both the cognitive area, including seven domains such as self-image, symbolic play, object-relation schemata, operational causality, means–ends relations, spatial relations and object permanence, and the socio-emotional area, including nine domains such as behavior regulation, social interaction, joint attention, expressive language, receptive language, vocal imitation, gesture imitation, affective relations, and emotional expression. Based on the Piaget and Fisher models of child development (56, 57), this assessment tool determines the developmental level in each of 16 domains, according to a hierarchical list of items for each developmental level: level 1 (4–8 months), level 2 (8–12 months), level 3 (12–18 months), and level 4 (18–24 months). Each item was rated, either as grade 2 (= complete success), grade 1 (= emergence or relative success with a bit of help and a demonstration), or grade 0 (= failure in spite of some help and a demonstration). The developmental level reached by the child in a domain corresponds to a level in which at least one of the items among the higher level was graded 1. A developmental level score from 1 to 4 was determined for each of the 16 domains, and this provides a developmental profile for each child (**Figure 1**).

The overall average levels, as well as both cognitive and socio-emotional development and indices of heterogeneity of profile for overall, cognitive and socio-emotional domains, were calculated. These indices corresponded to the mean difference (in absolute values) between all the level scores (1–4) of each domain multiplied by 10. They ranged from 0 (no heterogeneity) to 16 (maximum heterogeneity). An example of index calculation is presented in **Table 2**.

**Table 2:** An example of the calculation of Heterogeneity Index: for the Cognitive Heterogeneity Index (CHI).

Domains	Cognitive	OP	SR	ME	OC	Sch	SP	SI	Sum of differences (Σ) In absolute value
Cognitive	Levels	2	3	2	3	2	3	2	
OP	2		1	0	1	0	1	0	3
SR	3			1	0	1	0	1	3
ME	2				1	0	1	0	2
OC	3					1	0	1	2
Sch	2						1	0	1
SP	3							1	1
SI	2								
						Total			12
						Mean = Σ ÷ 21			12 ÷ 21 = 0.57
						CHI = MOY × 10			0.57 × 10 = 5.7

Cognitive, cognitive domains; OP, object permanence; SR, spatial relations; ME, means–ends; OC, operational causality; Sch, object relation schemata; SP, symbolic play; SI, self-image.

The transformation of the development level score into a developmental quotient (DQ) is based here on two empirical findings, namely, the existence of very high correlations on the one hand between the scores (not corrected for unreliability) of the SCEB and those of Brunet–Lézine Revised (52) for both a sample of children with a diagnosis of ASD [r(91) = 0.87, p < 0.001] and an international sample of young typical children [r(71) = 0.90, p < 0.001], and on the other hand, between SCEB scores and the chronological age observed for a sample of young typical French children [*r*(104) = 0.94, *p* < 0.001] and of young typical Brazilian children [*r*(20) = 0.70, *p* < 0.0001] (unpublished data). As no Algerian children of this study were tested with the Brunet–Lézine Scale and no typical young Algerian children were tested with the SCEB, correlations between these variables could not be calculated. However, as the correlations’ coefficients for children with ASD and for international samples of young typical children were high, it was assumed that these relationships might be applied to this group of children.

These observations rely on different processes to determine a developmental quotient from a SCEB overall developmental score: 1) according to the parameters (slope and intercept) of the regression equation between the sum of 16 SCEB developmental scores and the Brunet–Lézine Developmental Age (the equation is the following: developmental age, DA (in month) = 0.414 × the sum of the scores on SCEB scales + 2.21) (47) or 2) by simply rescaling the SCEB scores (level 0–4) on a scale from 0 to 24 months (ratio, 6). The DAs, as defined with the equation or rescaling, are perfectly correlated. This DA is divided by the chronological age (CA) in months. The product of this ratio is then multiplied by 100 to obtain a developmental quotient [DQ = (DA/CA) × 100]. It is this last possibility that is being applied in the current study. Thus, Development Quotients are: overall scores SCEB (0–4) rescaling on 0–24 (months) (DA) divided by chronological age (CA) and multiplied by 100 (**Table 3**).

All scores of the SCEB presented appropriate reliability and validity according to the usual psychometric criteria (34, 47).

**Table 3:** Mean overall level of development (SCEB) and development quotients and CARS scores of children with ASD by country and of children with typical development (TD).

Country	Overall level of development		Development quotients		CARS scores			
	Mean	SD	Mean	SD	Mean	SD	Min	Max
Algeria *N* = 39	2.73	0.91	24.58	7.22	42.56	7,22	30	57
Brazil *N* = 40	3.11	0.75	38.52	6.08	37.89	6,08	30	56
France *N* = 40	2.66	0.46	34.24	3.33	36.95	3,33	30	42.5
Total (ASD) *N* = 119 (101 male; 18 female)	2.83	0.75	32.51	6.22	39.11	6.22	30	57
TD *N* = 40	2.97	0.88	119.97	14.93	–	–	–	–

The mean sample score of ASD groups on overall level of development was 2.83 corresponding to the 12–18 months development stage. On Developmental Quotients, it was 32, corresponding to severe ID, and on the CARS scale, it was 39.00, which falls within the “severely autistic” degree (**Table 3**).

There were no significant differences between the TD and the ASD groups for number of subjects per gender [χ²(1, *N* = 159) = 3.07, *p* = 0.079], country [χ²(2, *N* = 159) = 0.03, *p* = 0.985], and the overall development level score on SCEB [*F*(1, 157) = 0.94, *p* = 0.334].

Some differences (two-sided *p* value) were noticeable between ASD groups: the CARS score [*F*(2, 115) = 10.3, *p* < 0.001], the developmental quotient [*F*(2, 115) = 8.9, *p* < 0.001], and the overall level of development (SCEB) [*F*(2, 115) = 4.15, *p* = 0.02]. There was no significant sex difference for age [*F*(1, 117) = 1.38, *p* = 0.243], CARS scores [*F*(1, 117) = 0.31, *p* = 0.578], overall levels of development [*F*(1, 117) = 1.05, *p* = 0.308], or developmental quotients [*F*(1, 117) = 0.85, *p* = 0.359].

### Procedure

Each participant was accompanied by his/her parents to the medical or psychological clinical service in order to perform the examination. The child was examined in a single 30–45-min session in a suitable room by a psychologist experienced with children presenting ASD and ID and familiar with the SCEB material. The SCEB was administered in the same naturalistic manner to all participants, and assessment protocols were respected at all times. As such, different children had comparable experiences during testing. There was no strict order for the presentation of the material, and the examiner chose toys and objects according to child’s interest. With patience and determination, he/she captured the child’s attention by showing the material (one activity at a time) and by inviting the child to use it. The psychologist interacted with the child by inviting him/her to make verbal and nonverbal contact with him/her and to manipulate the test material. He/she observed the child’s behaviors, including response and initiating interactions, objects and toy manipulation, vocalizations, words and sentences of two or more words, facial expressions, imitation, and joint attention behaviors. Since each child’s assessment session was videotaped, the examiner was able to review the recordings in order to rate the items corresponding to the observed behaviors. Complementary information was obtained from the family or educators after the examination, in particular for the Emotional Expression and Affective Relations domains. In fact, these domains included behaviors mainly expressed in the presence of parents or teachers, such as “He/she can recognize and differentiate between his/her parents” or “He/she delights in provoking the favorite person.”

The study protocol resulted in the establishment of research agreements with universities and specialized clinical services for the assessment and/or psycho-educational support of children with autism. The retrospective data collection from experienced psychologists, already specifically trained to use the SCEB instrument, was carried out in France. Concerning the recruitment and assessment of other children in the clinical population (in Brazil and Algeria), research university collaborations were developed in the use of the SCEB in clinical services in each country. Before we started the study in Algeria and Brazil, and to control for the cross-cultural contexts, we ensured that the SCEB was easily usable for all children regardless of their culture. As a first step, psychologists from each of these countries assessed a few children with ASD and then were able to confirm that the SCEB could be easily and correctly used without adaptation. On the one hand, the material is very common and familiar to children (toys such as cars, dolls and blocks…), and on the other hand, the vocal, verbal (e.g., “give me the car,” “look at pictures,” “show me the ball”), and nonverbal instructions (e.g., “point to a picture or an object,” “imitate a gesture of clapping,” “look at the mirror,” “hide an object under a box”) are simple and very accessible to and understood by children across these cultures. Moreover, psychologists were specifically trained (individual and group seminars directed by the first and last authors) on the use of the SCEB and continuously supervised throughout the duration of the research (ratings of SCEB sessions from videotapes). Double ratings of five Brazilian and five Algerian SCEB protocols were made by the first author to obtain a complete interagreement for each (99%). Each of these five protocols was initially rated respectively by the Brazilian and Algerian psychologists who had examined the child using the SCEB in his/her native country’s clinical service. The second rater (first author) then watched the videotapes and rated the child’s behaviors independently in the Laboratory of Psychopathology and Health Processes (Paris). During these double ratings, the Brazilian or the Algerian psychologist was present during viewings with instructions to help only for the understanding of verbal words and sentences the child pronounced. The two domains of Emotional Expression and Affective Relations could not be double-rated because their items were mainly rated through information from parents or teachers. However, the information was discussed by both psychologists and approved by the second rater who could then validates the rating of items and the scoring of developmental levels.

### Analysis

Developmental levels on the 16 SCEB scales correspond to developmental age periods: level 1 (4–8 months), level 2 (8–12 months), level 3 (12–18 months), and level 4 (18–24 months). Nevertheless, for the present samples, the distributions of the SCEB scores are not random samples of a Gaussian distribution. This is not the case with the overall level of development. The heterogeneity index, CARS score, overall level of development, Developmental Quotient, and age are continuous variables, and the Kolmogorov–Smirnov test shows no significant difference with normal distribution. No outliers were observed that were more than 3.5 standard deviations away for each group. When the variables could not be considered as normally distributed continuous variables, nonparametric tests were used such as the Spearman rank correlation, the Wilcoxon signed-rank test that is a nonparametric test equivalent to the dependent *t*-test, and also the Friedman rank sum test (58). The latter is a nonparametric alternative to the one-way ANOVA with repeated measures. It is used to test for differences between repeated measures when the dependent variable being measured is ordinal. It can also be used for continuous data that has gone against the assumptions necessary to run the one-way ANOVA with repeated measures. Pairwise comparisons were made with probability adjustment for the number of tests according to the Bonferroni method. Data analyses were performed with R Development Core Team Software (59). The comparison of heterogeneity levels of development profiles between ASD and TD groups uses global, cognitive, and socio-emotional heterogeneity indices and is also based on a more basic level of information. The most detailed information is obtained, group by group, with the identification and counting of all significant differences that appear for 120 possible comparisons [(16 × 15)/2] between the scores on the 16 SCEB scales (within-subjects factor). The number of significant differences (according to an alpha threshold of .05) is then compared between ASD and TD groups. The identification of the functions that collect the lowest and the highest scores makes it possible to search for regularities according to country.

Analysis steps

1. We first tested the hypothesis of differences between ASD and TD groups on the variability on SCEB scores (within-subjects factor). We assume that the number of significant differences between the scores on the 16 scales will be greater for the ASD groups than for the TD group.
2. We checked for regularity between three ASD groups regarding development scales that show the lowest and highest levels. We postulate that some differences will be noticeable in a few cognitive and socio-emotional domains.
3. We tested the null difference hypothesis between groups from different countries in mean indices of heterogeneity profiles, but significant differences are expected between the ASD and TD groups.

In order to test this assumption, a hypothesis of no difference in indices of heterogeneity profiles by country was tested using analysis of variance. There was an overall index of heterogeneity for everyone. The difference between heterogeneity indices on the “cognitive” and “socio-emotional” scales was also computed; a positive value of this difference for a given subject indicates greater heterogeneity in cognitive aspects than in socio-emotional aspects. The range of the socio-emotional and cognitive heterogeneity scales was the same. A difference between socio-emotional and cognitive heterogeneity is relevant, and intergroup analysis with a single dependent variable (score difference) was performed because it is simpler and more powerful (the difference score reduces intragroup variability) than an interaction analysis with two dependent variables (within variable) and an intermediate variable.

4. We tested the hypothesis of no differences between groups in difference of cognitive and socio-emotional heterogeneity indices. We expected no significant difference between all the groups.
5. We checked if there was a relationship among heterogeneity, chronological age, degree of severity of autism (CARS), degree of severity of ID (Development Quotient), and overall level of development. We assumed significant correlations for ASD groups but no significant relationship between heterogeneity, chronological age, and overall level of development for the TD group. It was expected that correlation coefficients would be similar according to nationality for the ASD groups.
6. We tested the hypothesis of a relationship between heterogeneity indices, each domain’s developmental levels, and the severity of autistic symptomatology.

## Results

### Testing the Hypothesis of Differences Between the ASD and the TD Groups Corresponding to Differences on the SCEB Scores (Within-Subjects Factor)

**Figure 2:**
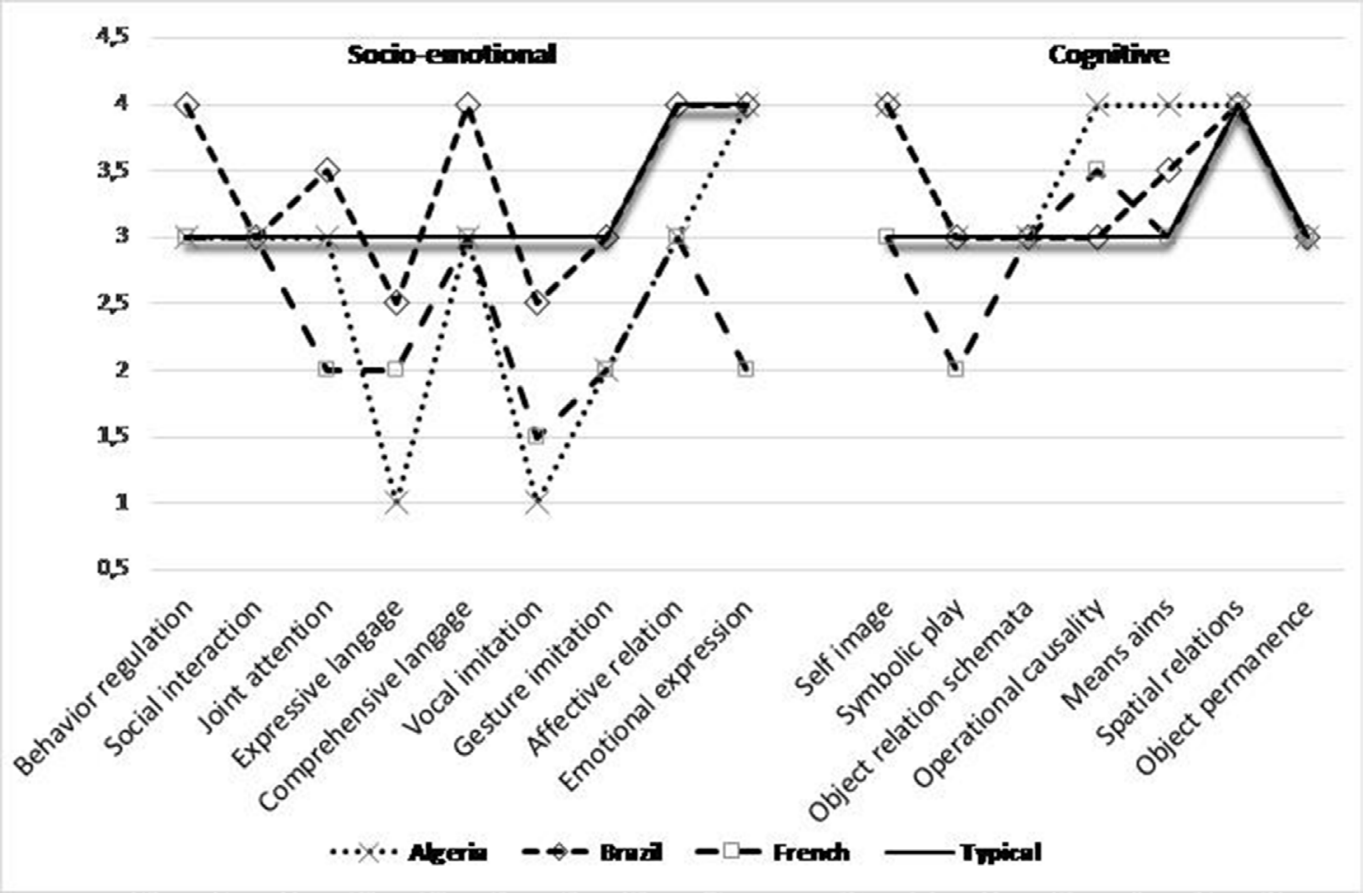
Profiles of median developmental level scores (from 1 to 4) in all the 16 cognitive and socio-emotional domains on SCEB, for the three groups and for French children with ASD as well as the typical development groups from Algeria and Brazil. *Legend*: Socio-emotional domains: BR, Behavior Regulation, SI, Social Interaction, JA, Joint Attention, EL, Expressive Language; RL, Receptive Language; VI, Vocal Imitation; GI, Gestural Imitation; AR, Affective Relation; EE, Emotional Expression. Cognitive domains: SI, Self-Image; SP, Symbolic Play; Sch, Object relation schemata; OC, Operational Causality; ME, Means–Ends; SR, Spatial Relations; OP, Object Permanence.

A comparison of differences on the 16 domains SCEB scores was conducted independently for the four groups. Then, the number of cases in which a difference was observed was compared between the groups. Median developmental level scores in domains on the SCEB for four groups are presented in **Figure 2.**

The results show significant differences between scores on the 16 SCEB domains for each group, with lower effect for the typical group [typical: Friedman χ²(15, *N* = 40) = 90.41, *p* < 0.001; Algeria: Friedman χ²(15, *N* = 39) = 162.01, *p* < 0.001; France: Friedman χ²(15, *N* = 40) = 244.27, *p* < 0.001; Brazil: Friedman χ²(15, *N* = 40) = 150.83, *p* < 0.001].

The results of the pairwise comparisons (*n* = 120) using the Wilcoxon signed-rank test with a probability adjustment (for the number of tests) according to the Bonferroni method are used to compare the number of significant differences [alpha (two-sided): 0.05] between the SCEB scores between the groups. The number of cases in which a difference was observed was compared between the groups using the χ² test (**Table 4**).

**Table 4:** Size of significant (α = .05) and insignificant differences on 16 SCEB scores by group.

Group	n.s.	Significant differences
Algeria	82	38
χ² components	0.23	0.60
Brazil	83	37
χ² components	0.14	0.37
France	69	51
χ² components	3.54	9.14
Typical	112	8
χ² components	7.52	19.41

n.s, non-significant.

The χ² in the table shows significant variations [χ² (3, *N* = 480) = 40.96, *p* < 0.001]. The main contributions to χ² appear with the typical group, as expected, with a lower number of significant differences, while the French group showed the highest number of significant differences. The heterogeneity of profiles is therefore much more significant for all of the ASD groups than for the TD group of children.

### Testing the Hypothesis of No Differences Between Developmental Levels in All 16 Domains

The results of the pairwise comparisons (two-tailed probability of a *Z* value of Wilcoxon signed-rank test adjusted with the Bonferroni method) showed some regularity in the three ASD groups. The lowest average developmental levels were systematically observed in the Expressive Language and Vocal Imitation domains. Thus, they differed more significantly (α < 0.05) than other SCEB domains.

— In the Algerian group, Expressive Language differed significantly from all the other domains except for Vocal Imitation, Gestural Imitation, and Receptive Language; Vocal Imitation did not differ from the Expressive Language and Gestural Imitation domains.
— In the French group, Expressive Language differed significantly from 7 of the other domains, and Vocal Imitation differed from 9 of the 15 other domains.
— In the Brazilian group, Expressive Language differed significantly from 6 of the other domains, and Vocal Imitation differed from 5 of the 15 other domains.

The domains that showed the highest developmental levels varied according to the groups, but they always involved the cognitive area, with Means–Ends in the Algerian group (differing significantly from 6 of the other domains), Operational Causality and Spatial Relations in the French group (differing respectively from 11 of the other domains), and Spatial Relations also in the Brazilian group which differed significantly from 11 of the other domains.

### Testing the Hypothesis of No Differences Between Groups in Mean Indices of Heterogeneity Profiles

ANOVA results on the overall scores of heterogeneity indices of the four groups (**Table 5**) showed that there were significant differences *F*(3, 155) = 17.9, < 0.001). Pairwise comparisons using *t* tests with a *p* value adjusted with the Bonferroni method showed differences between each ASD group and the TD group [Algeria: *t*(77) = 6.57, *p* < 0.001; Brazil: *t*(78) = 4.26, *p* < 0.001; France: *t*(78) = 8.63, *p* < 0.001]. However, the results also showed a significant difference between the Algerian and the Brazilian ASD groups [*t*(77) = 2.29, *p* = 0.025].

**Table 5:** Overall, cognitive, and socio-emotional heterogeneity indices of the three ASD groups and the TD group.

Groups	Overall heterogeneity		Cognitive heterogeneity		Socio-emotional heterogeneity	
	Mean	SD	Mean	SD	Mean	SD
Algeria (*n* = 39)	10.95	5.59	7.25	5.71	10.65	5.73
Brazil (*n* = 40)	9.49	2.45	8.07	2.77	9.07	3.63
France (*n* = 40)	8.25	4.89	7.97	4.37	8.42	6.64
Total ASD group (*n* = 119)	9.55	4.60	7.77	4.41	9.37	5.51
TD group (*n* = 40)	4.47	2.74	4.64	2.81	3.76	3.59

### Testing the Hypothesis of No Differences Between Groups Regarding the Difference of Cognitive and Socio-Emotional Heterogeneity Indices

The analysis of variance showed a significant difference between the groups [*F*(3, 155) = 4.74, *p* = 0.003]. Pairwise comparisons using *t* tests with *p* value adjusted with the Bonferroni method showed a difference only between the Algerian ASD group and the TD group [*t* (77) = −3.68, *p* = 0.002].

### Testing the Relationship Between Heterogeneity, Chronological Age, Degree of Severity of Autism, Degree of Severity of Intellectual Disability, and Overall Level of Development

To estimate the homogeneity of correlations between heterogeneity and chronological age, degree of severity of autism, degree of severity of ID, and overall level of development across groups, we considered the existence of overlaps between the confidence intervals (**Table 6**). The analysis did not indicate significant differences in correlations except between the French ASD and TD groups for the overall developmental level and between both the Algerian and Brazilian ASD and TD groups for the DQ.

**Table 6:** Correlations between heterogeneity, chronological age, degree of severity of autism, degree of severity of intellectual disability, and overall level of development by groups.

Country	Criteria	Heterogeneity	Adjusted two-sided *p* values (Holm’s method)	C.I. [0.95] of Pearson *r*	
		Pearson *r*		Lower	Upper
Algeria	Age	−0.38	0.064	−0.62	−0.08
Brazil		0.05	1.000	−0.27	0.36
France		−0.16	1.000	−0.45	0.16
Typical		−0.34	0.103	−0.59	−0.03
Algeria	CARS	0.48	0.014	0.19	0.69
Brazil		0.66	<0.0001	0.44	0.80
France		0.46	0.027	0.17	0.67
Algeria	Overall level of development	−0.80	<0.0001	−0.89	−0.65
Brazil		−0.88	<0.0001	−0.93	−0.78
France		−0.44	0.036	−0.66	−0.15
Typical		−0.20	0.212	−0.48	0.12
Algeria	DQ	−0.26	0.228	−0.53	0.06
Brazil		−0.63	<0.0001	−0.79	–0.40
France		0.02	1.000	−0.29	0.33
Typical		0.44	0.020	0.14	0.66

Moreover, we noted that heterogeneity indices were not related to chronological age. However, there is evidence of significant relationships between developmental profile heterogeneity indices and both the severity of autistic symptomatology, assessed with CARS, and overall developmental level assessed with the SCEB (negative relationship), except for the TD group. There is also a negative and significant correlation between the degree of severity of ID (DQ) and the heterogeneity, for the Brazilian group, and a positive and significant correlation for the TD group.

### Testing the Relationship Between Heterogeneity Indices and Developmental Levels of Each Domain and the Severity of Autistic Symptomatology

In **Table 7**, we can note Spearman’s rank correlations show that overall heterogeneity links are the closest to verbal skills: Vocal Imitation, Receptive Language, and Expressive Language in ASD children. We also observe moderate links between heterogeneity and these same skills in DT children: Expressive Language and Vocal Imitation, and the cognitive domain: Operational Causality.

**Table 7:** Spearman’s rank correlations between heterogeneity and overall level of development in the 16 SCEB domains for the ASD and the TD group and CARS scores for the ASD group.

	Heterogeneity (global)		CARS scores
	ASD	TD	ASD
Behavior Regulation	−0.34^***^	−0.18	−0.21^*^
Social Interaction	−0.65^***^	−0.22	−0.40^***^
Joint Attention	−0.58^***^	−0.16	−0.31^***^
Expressive Language	−0.79^***^	−0.48^**^	−0.50^***^
Receptive Language	−0.80^***^	−0.23	−0.52^***^
Vocal Imitation	−0.85^***^	−0.54^***^	−0.43^***^
Gestural Imitation	−0.75^***^	−0.21	−0.44^***^
Affective Relation	−0.25^**^	−0.11	−0.23^*^
Emotional Expression	−0.49^***^	−0.05	−0.22^*^
Self-Image	−0.34^***^	−0.18	−0.25^**^
Symbolic Play	−0.48^***^	−0.21	−0.22^*^
Object Relation Schemata	−0.51^***^	−0.22	−0.40^***^
Operational Causality	−0.34^***^	−0.32^*^	−0.34^***^
Means–Ends	−0.32^***^	−0.21	−0.08
Spatial Relations	−0.17	−0.10	−0.12
Object Permanence	−0.28^**^	−0.23	−0.28^**^

Two-sided p values: *p < 0.05, **p < 0.01, ***p < 0.001.

We also note that the closest links between CARS scores and SCEB domains occur in verbal skills, Expressive Language, Receptive Language, and Vocal Imitation, but they do not occur at all in Spatial Relations and Means–Ends.

## Discussion

The present study investigated whether the cognitive and socio-emotional developmental profile of children presenting ASD with comorbid ID is different from that of a group of young typical children. Moreover, we sought to identify the skills that had the lowest and the highest developmental levels and to explore whether the heterogeneity of the profiles in children with ASD is a common characteristic (intraheterogeneity), regardless of the country of origin (interheterogeneity), but is correlated to the severity of autistic symptomatology and intellectual disability and to the overall development level.

Results showed that children with ASD and ID developmental profiles were heterogeneous as opposed to typical children in all the developmental heterogeneity indices. Overall, cognitive, and socio-emotional were significantly higher than in the typical group of children. These findings confirm Bernard’s results (42) on a larger group of 119 typical children from seven countries on three continents (America, Europe, and Africa). In fact, the low heterogeneity mean indices and the limited differences between developmental levels of all the cognitive and socio-emotional domains confirm that children with typical development have a cognitive (60–62) and social-communicative (63, 64) homogeneous developmental profile. In children with ASD, atypical developmental patterns were mainly explained by relative weaknesses and developmental delays in some abilities such as joint attention, expressive and receptive language, and symbolic play (23, 25–27, 28, 65).

Moreover, we found a similar cognitive and socio-emotional development intraheterogeneity in children with ASD and ID from samples in three different countries but only a few moderate differences between them that affected mean heterogeneity indices profiles between the Algerian and Brazilian ASD groups. This heterogeneity was also evidenced by several differences between SCEB developmental level scores in ASD that turned out to be even more numerous in the French group and not only in cognitive areas but also in socio-emotional areas. Significant differences were found for Expressive Language (EL) and Vocal Imitation (VI) median developmental levels, which were the lowest. The EL and VI median developmental level scores (from 1 to 2.5 = 8–12 months of age) correspond to the prelinguistic phase when the infant can only imitate his/her own vocal productions and express monosyllabic (“ba”) and bisyllabic (“tata”) sounds and a few new sounds and when he/she begins to pronounce one or two words (“dad,” “mum”). Language and vocal imitation deficits may be related to motor difficulties (66), to early oro-motor anticipation deficits (67), and to very early atypical vocal productions (68, 69). They may impact social communication (21) and can be explained by early hypoactivity in language-sensitive superior temporal cortices implying different outcomes for language, which is poorer in preschool children with ASD (70). Moreover, cognitive domains demanding mainly nonverbal actions, such as using means to reach a goal or establishing causal and spatial relationships between objects, showed better developmental levels compared to expressive language and vocal imitation domains. Thus, these domain-specific developmental strengths and weaknesses might reflect the developmental heterogeneity corresponding to the nonverbal and verbal discrepancy noted in the most intellectually disabled children with ASD presenting the lowest verbal development levels (31).

In addition, there is evidence of some deficits and delays in verbal expression, which are also known to be present in toddler siblings of children with ASD (71). They are predictors of ASD and later-diagnosed ASD in infants at high risk, even though the social communication developmental pathways are variable during the 6–36-month period (72). These linguistic deficits that were common in children with other neurodevelopmental disorders without social communication deficits, such as specific language impairment (73) and intellectual disability with genetic syndromes (74, 75), might be related to common cerebral developmental deficits or dysfunctions (70, 76, 77).

Unexpectedly, a positive correlation between heterogeneity and DQ was observed in the typical group of young children. The typical children’s mean DQ was superior to the normative benchmark of 100. This may be due to sampling bias given that the recruitment of typical children was done from among psychologists’ acquaintances. Thus, for children with the most advanced development in our sample, there is a developmental heterochrony.

This absence of statistical differences between cognitive and socio-emotional heterogeneity indices in any of these three ASD groups shows a pattern across the three countries, although in the Algerian group, the difference between these indices appears significantly higher than in the typical group, showing a higher developmental intraheterogeneity. This could be explained by a high socio-emotional heterogeneity in this group of children, being reflected by lower developmental levels in both Expressive Language (=1) and Vocal Imitation (=1) domains. In a transcultural cross-sectional study (39), two samples of autistic children were compared in two large Arab countries: Egypt (*N* = 20) and Saudi Arabia (*N* = 28). With regard to the behaviors and development of both groups assessed with Gilliam subscales (78), although there were significant differences in the stereotypical and developmental characteristics (Saudi children showing significantly more stereotypical and lower developmental abilities than Egyptian children), there was no significant difference between both groups regarding level of intelligence. Moreover, the Saudi group showed significantly more severe and profound communication defects as assessed with the Vineland communication subscale.

Furthermore, overall developmental heterogeneity was positively correlated with the degree of severity of autism (CARS score) and negatively correlated with the level of development for all ASD groups. Thus, the higher the developmental heterogeneity, the more severe the autistic symptomatology and the lower the overall developmental level are. A significant relationship between the degree of severity of ID (DQ) and heterogeneity was only observed for the Brazilian group. This specific relationship for the Brazilian group might be explained by intergroup age differences, the severity of autism, and the level of development, given that correlations between heterogeneity with ID controlled by differences in age, severity of autism, and levels of development were not significant for each group. Thus, these results indicate that overall developmental heterogeneity was not an effect of severity of ID but rather a characteristic of the development of children with ASD. This seems to prove the universality of this atypical development in children with ASD and comorbid ID, which is characterized by socio-cognitive developmental heterogeneity correlated to linguistic function delay, for example, in expressive and receptive language and vocal imitation.

Furthermore, while this correlation was also noted in typical children, the link was higher in ASD children and was even more intense in children with severe autistic symptomatology and with low levels of development in these language skills. The results observed in children with ASD and severe ID (25 < DQ < 35), low developmental levels (4–24 months) and important differences in chronological age (1 year and 9 months to 14 years) confirm results obtained in children with ASD without ID by Joseph et al. (30) and Ankenman et al. (31). In fact, these researchers noted that the cognitive heterogeneous profile characterized by the discrepancy between nonverbal > verbal abilities was related to a high level of autistic symptomatology and that it was higher in low developmental level children. Thus, while their manipulations and spatial skills grow with age, their functional language does not develop, so the gap between these abilities and intraindividual heterogeneity increases. In a longitudinal study, Vivanti et al. (79) showed that the more “autism specific” symptoms young children have, the more at risk they are of poor cognitive outcome. In addition, Baghdadli et al. (11) found that the developmental trajectory of socialization and communication disorders in children with ASD was associated with the worst outcomes in lower functioning children, with absence of language abilities, higher severity of autistic symptoms, and lower levels of cognitive functioning related to objects and to people.

Our result shows that heterogeneity, which is observed at the individual level, is also found at the normative level. This indicates that heterogeneity at the individual level is not random and is a true marker of the development of children with ASD. This heterogeneity appears to be stable whatever the country of origin. Thus, at the interheterogeneity level, it was shown that the SCEB function rankings according to the developmental levels are relatively similar across cultural groups, as shown by between-group correlations.

This study was essentially empirical, descriptive, and exploratory. Although psychological assessment with the SCEB is founded on theoretical and well-known models of cognitive and socio-emotional development in young children (56, 57) and based on robust data on cognitive and communicative skills in children with ASD and ID (23–27), the absence of theoretical models of typical psychological developmental cross-cultural differences prevented us from developing psychological and/or social explanations of potential differences or similarities in children with atypical development such as those presenting ASD. These differences and similarities might be explained by more specific variables, for example, the genetic, biological, cerebral, and developmental trajectory (10, 11, 70, 76) characterizing each child with ASD. Further cross-national studies should be carried out on these variables to investigate the presence and/or absence of differences and similarities in developmental profiles between children with ASD from various countries (38, 39).

No great variability in the cognitive and socio-emotional developmental profiles of children with ASD and ID was found as a function of their culture of origin. This result confirms the relevance of comparative and intercountry studies dealing with neurodevelopmental disorders such as ASD and the development of assessment instruments adapted both to this clinical subgroup of children with ASD and severe ID as a comorbidity and to each country. Some studies have already been carried out on this topic with the SCEB in other countries, such as Belgium (35) and Italy (80, 81).

Moreover, given that the developmental heterogeneity in children presenting with ASD, severe ID, and low developmental levels in expressive language and vocal imitation skills has been shown to be a major feature across these countries, this dimension must be assessed and analyzed, mainly to facilitate the development of early and appropriate interventions that are focused on these specific disabilities and are based on cognitive and socio-communicative developmental profiles. It is also important to involve the parents in early intervention (82–86) in order to decrease adaptive impairments that are mainly explained by socio-communicative ASD symptoms severity (87).

## Author’s Note

This work was carried out based upon four doctoral theses, from FA (88), LB (89), M-AB (42), and CM (90).

## Author Contributions

Supervision and double rating of videotapes: M-AB, J-LA. Developmental and quantitative diagnostic data collection: M-AB, CM, MN, AS, FA, MG, RB, KK, NS, LB, LF, YC, CB, FB-B. Study design: J-LA, M-AB, ET, CM, MC. Data analysis: ET, J-LA, M-AB. Writing: J-LA, M-AB, ET, KK, MC, CM, JW.

## Funding

Funds from IDEX of Sorbonne Paris City University making researchers’ international mobility between France and Brazil possible (2014–2018).

## Conflict of Interest Statement

J-LA is the author of the SCEB edited in the Pearson France-ECPA.

The remaining authors declare that the research was conducted in the absence of any commercial or financial relationships that could be construed as a potential conflict of interest.

## Footnotes

1 Order no 2017-884 of 9 May 2017 modifying some regulatory dispositions relative to research implying human people. *Official Journal of French Republic*, 10 May 2017, Text 84 on 396.

2 Universitary Ethic and Deontological Chart. *Superior Teaching and Research Ministry*, 2010. Algeria.

3 Process No. 306.264, National Commission for Research Ethics (CONEP). *Brazilian Ministry of Health*, 2015, Brazil.
